# Epidemiology and Clinical Manifestation of West Nile Virus Infections of Equines in Hungary, 2007–2020

**DOI:** 10.3390/v14112551

**Published:** 2022-11-18

**Authors:** Orsolya Eszter Fehér, Péter Fehérvári, Csenge Hanna Tolnai, Petra Forgách, Péter Malik, Ákos Jerzsele, Zsombor Wagenhoffer, Otto Szenci, Orsolya Korbacska-Kutasi

**Affiliations:** 1 Institute for Animal Breeding, Nutrition and Laboratory Animal Science, University of Veterinary Medicine, István utca 2, 1078 Budapest, Hungary; 2 Department of Biomathematics and Informatics, University of Veterinary Medicine, István utca 2, 1078 Budapest, Hungary; 3 Centre for Translational Medicine, Semmelweis University, 1085 Budapest, Hungary; 4 University Equine Clinic, University of Veterinary Medicine Vienna, Veterinärplatz 1, 1210 Wien, Austria; 5 Department of Microbiology and Infectious Diseases, University of Veterinary Medicine, Hungária Krt. 23-25, 1143 Budapest, Hungary; 6 National Food Chain Safety Office, Veterinary Diagnostic Directorate, Tábornok u. 2., 1143 Budapest, Hungary; 7 Department of Pharmacology and Toxicology, University of Veterinary Medicine, István utca 2, 1078 Budapest, Hungary; 8 National Laboratory of Infectious Animal Diseases, Antimicrobial Resistance, Veterinary Public Health and Food Chain Safety, University of Veterinary Medicine Budapest, István utca 2, 1078 Budapest, Hungary; 9 Department of Obstetrics and Food Animal Medicine Clinic, University of Veterinary Medicine, István utca 2, 1078 Budapest, Hungary

**Keywords:** West Nile virus, Equidae, epidemiology, endemic, emerging, West Nile neuroinvasive disease

## Abstract

West Nile virus (WNV) is an emerging pathogen in Hungary, causing severe outbreaks in equines and humans since 2007. The aim of our study was to provide a comprehensive report on the clinical signs of West Nile neuroinvasive disease (WNND) in horses in Hungary. Clinical details of 124 confirmed equine WNND cases were collected between 2007 and 2019. Data about the seasonal and geographical presentation, demographic data, clinical signs, treatment protocols, and disease progression were evaluated. Starting from an initial case originating from the area of possible virus introduction by migratory birds, the whole country became endemic with WNV over the subsequent 12 years. The transmission season did not expand significantly during the data collection period, but vaccination protocols should be always reviewed according to the recent observations. There was not any considerable relationship between the occurrence of WNND and age, breed, or gender. Ataxia was by far the most common neurologic sign related to the disease, but weakness, behavioral changes, and muscle fasciculation appeared frequently. Apart from recumbency combined with inappetence, no other clinical sign or treatment regime correlated with survival. The survival rate showed a moderate increase throughout the years, possibly due to the increased awareness of practitioners.

## 1. Introduction

West Nile virus (WNV) is a globally emerging pathogen belonging to the genus *Flavivirus,* in the family *Flaviviridae*. The arthropod-borne single strained RNA virus is a member of the *Japan encephalitis virus complex* and has a strong genetic relationship with other high-risk human and animal pathogens, such as tick-borne encephalitis virus (TBEV) and Usutu virus (USUV) [1,2]. WNV was first detected in the West Nile province of Uganda in the mid-1930s, and since its first appearance, it has caused diseases with a wide range of signs, from asymptomatic seropositivity, through flu-like fever illness, to severe neurological diseases in humans, horses, and birds. WNV is known as the most widespread *Flavivirus* pathogen in the world, causing severe outbreaks worldwide, with thousands of human and animal cases [1]. *Culex* mosquitos are the main arthropod vectors of WNV, but other types of transmission, such as blood transfusion or organ transplantation, can occur [2,3,4]. Due to the activity of mosquitoes as vectors, there can be seasonality of infections in most countries with temperate climates. Weather conditions, especially rainfall and humidity, can strongly influence mosquito reproduction, which can increase the number of cases in a region and change the previous seasonal occurrence of infections [5].

Humans and horses are considered dead-end hosts of the virus, as in these species the viremia phase is low and short, and mosquitos rarely become infected. There is a wide range of species of birds in which high levels of viremia can develop, and these may act as transmission species [6].

In Hungary, WNV caused the first outbreak in 2003 in a flock of geese, with another in 2004 in goshawks [7]. In Hungarian horses, WNV infection was first diagnosed in the autumn of 2007, and equine neuroinvasive cases have occurred every year since, with the number of cases varying widely. In the avian outbreak in 2003, lineage 1 strain was identified, with a strong genetic relationship to the strains isolated in 1998 in Israel and in 1999 in North America [8]. Lineage 1 strains are responsible for the outbreaks in the western hemisphere [9]. Since the 2004 outbreak in goshawks, all further Hungarian West Nile neuroinvasive disease (WNND) outbreaks have been caused by lineage 2 WNV strains [7]. After the first Hungarian equine WNND outbreak in 2008 [10], genetically closely related lineage 2 strains have caused human, avian, and equine outbreaks in Greece (2010) [11], Italy (2011) [12,13], Austria (2016) [14], France (2020) [15], Germany (2018) [16], and recently in the Netherlands (2020) [17,18,19,20]. In previous serological survey studies in Hungary, in 2011, 25.7% [21]; in 2013, 14.63% [22] of the tested, non-vaccinated and apparently healthy horses appeared to be seropositive to WNV. After the 2018 outbreak season, in two unpublished serosurveys, we found 66.5% and 72.3% seropositivity among equids throughout the country. 

Approximately 1% of people infected develop flu-like symptoms or self-limiting febrile illness, and fewer cases end in the neurological form. Horses are more sensitive to WNV infection, and approximately 10% of the affected cases develop severe neurological signs [23,24,25]. Based on previous descriptions of lineage 1 outbreaks in Europe and North America, WNND in horses is mainly accompanied by clinical signs such as fever, depression, ataxia, paresis, tremors, or recumbence [11,24,26,27,28,29,30,31]. Although several descriptions of lineage 1 outbreaks have been published, less clinical reports on equine WNND caused by lineage 2 strains are available in the international literature [10,13,30,32,33,34]. In humans, a much more detailed classification of WNV-caused illnesses is available [25]. In humans, the infection can cause a flu-like syndrome called West Nile fever, which can be clearly differentiated from the neurologic forms of meningitis, encephalitis, or poliomyelitis [25]. In the case of horses, identification of specific neurologic forms is difficult because of the limited diagnostic modalities applied in these cases. 

Two types of commercial WNV vaccines are available in Hungary and prophylactic vaccination has been used in horses since 2010. Vaccination protocols follow the recent publications on both vaccines [22,35].

The aim of this retrospective case series study was to obtain an overview of the prevalence, seasonal and geographical distribution, and clinical manifestation of WNV infection in horses in Hungary.

## 2. Material and Methods

This retrospective case series study and outbreak description were based on observation and data collection in the period 2007–2020. 

We used the following diagnostic criteria based on the World Organisation for Animal Health (WOAH, founded as OIE), Manual of Diagnostic Tests and Vaccines for Terrestrial Animals 2013, Chapter 2.1.20, West Nile Virus (OIE Manual) [36]. We considered laboratory-confirmed recent West Nile virus infections, where WNV-specific IgM was detected in serum. Parallel neutralization tests detecting antibodies against WNV and other locally circulating flaviviruses (USUV and TBE) were only performed in a limited number of cases, and detailed results of neutralization tests are not included in this paper. Based on previous studies, the IgM capture ELISA used in our studies is more specific than other serological tests, so the misdiagnosis of secondary to cross-reactions should be negligible [37,38,39].

A clinical case of equine WNND was diagnosed if acute neurologic signs appeared during mosquito activity season and the horse tested positive for WNV-specific IgM in an officially certified laboratory. Sick horses were confirmed as clinical WNND cases if they met both criteria. According to laboratory cases, the only condition was the presence of the IgM antibody, but these horses were clinically asymptomatic.

### 2.1. Data Collection 

In Hungary, WNV infections and cases in any species require official notification of the Hungarian National Food Chain Safety Office (NÉBIH). Every horse with acute neurologic signs during the mosquito season must be tested for WNV infection at officially accredited laboratories. All of the participating laboratories were accredited and continuously re-evaluated by a foreign commission from the WOAH (founded as OIE).

For geographical and seasonal analysis, we used the database of the Hungarian National Food Chain Safety Office (NÉBIH) for all IgM-positive horses. For evaluation of WNND characteristics, we used symptomatic horses, which were diagnosed according to the WOAH guidelines, as described above. Attending veterinarians were contacted, to fill out a standard examination questionnaire (see Supplement Material Figure S1) about the confirmed WNND equine patients. 

The general data (age, breed, sex, usage) of the patient, the vaccination history, the onset and type of general and neurologic signs, sampling date, geographic origin, treatment, and outcome were recorded. The exact date of laboratory sampling was considered as the onset of the disease, as all blood samples were collected in less than five days after the appearance of the first clinical signs. 

### 2.2. Sample Collection 

Native blood samples were collected from the jugular veins of the horses and tested for the presence of IgM antibodies with a commercial Enzyme-linked Immunosorbent Assay (ELISA) (INgezim^®^ West Nile IgM, EUROFINS INGENASA, S.A, Spain) [15,38]. Equine veterinary practitioners carried out sample collection as part of their routine diagnostic work in seasonal neurologic cases. Samples were also submitted from horses without any clinical signs if the owner requested testing of stablemates.

### 2.3. PCR Analysis and Definition of Lineage 

Central nervous system (CNS) tissues of horses which were euthanized in clinical settings were examined using nested reverse transcriptase (RT)-PCR or real-time RT-PCR, to identify the viral pathogen, as described by Kutasi et al. [10].

### 2.4. Data Analysis

Two databases were used for our analysis. The first one contains the primary data (date of sampling, location, contact veterinarian) of all the ELISA IgM-positive horses from 2007–2020 in Hungary. The second database contained the general clinical data and clinical signs of the available horses from the first dataset. 

To assess temporal and spatial seropositivity patterns, we used annual descriptive statistics at a county level. To describe the relative frequency of clinical signs, we used the data of three years (2008, 2016, and 2018), where the most observations were available. We opted to not conduct hypothesis testing, as the data collection was not random and also potentially biased. Nonetheless, we present descriptive statistics, as the information and the patterns are of value. The relationship between clinical signs and survival was described using Classification and Regression Trees (CART) [40,41]. Briefly, these methods rely on a recursive algorithm to partition data based on explanatory variables, selecting the best splitting variable at each node. Here, we fitted a classification tree to the survival status of the individual, with all clinical signs and the presence/absence of data as explanatory variables.

## 3. Results

The temporal and geographical description of outbreaks was based on the data of 198 West Nile virus IgM positive horses. Not all of the horses tested positive showed clinical signs during testing, as some horses had a subclinical infection. Data of these horses were used for the evaluation of the geographical and temporary distribution. Out of the 198 IgM positive animals, we were able to collect the complete clinical history of 124 equids with seasonal acute neurologic signs, by contacting their treating veterinarians.

In the case of six euthanized horses, PCR screening was successfully carried out. In all of the samples, the lineage 2 strain was identified. We included one horse in our study that had an incomplete vaccination, with only one dose of vaccine before the onset of clinical signs. No other horse had a history of WNV vaccination prior to infection.

Table 1 contains the exact number of the detailed equine WNND cases during our study. The three highest number of cases in our study were collected in 2008 (16), 2016 (19), and 2018 (72).

### 3.1. Geographical Distribution 

**Figure 1:**
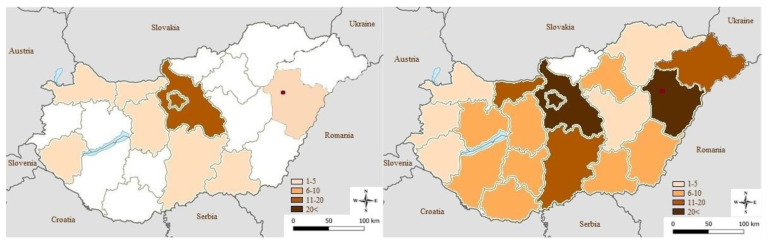
Geographical distribution in examination periods 2007–2013 and 2014–2020. Origin of the first detected equine case in 2007 is indicated by a red mark (Hortobágy, Hajdú-Bihar County).

The geographical distribution overview was divided into two 7 year examination periods: 2007–2013 and 2014–2020. In the first period, only a few counties reported equine cases of West Nile virus neuroinvasive disease (WNND). The origin of the first detected equine case, in 2007, was the region of Hortobágy, which is a part of the country that is well known for its migrating avian population [42]. The region of Hortobágy and county of Hajdú-Bihar remained severely infected during the later period as well. The virus mostly affected the whole country in the second examination period, with case numbers increasing significantly in many areas of Hungary. The results can be seen in Figure 1.

### 3.2. Temporary Distribution

When examining the seasonal occurrence in the years with the highest number of cases (2008, 2016, 2018), no major change in seasonality was detected, but a trend could be seen whereby the first cases occurred earlier in the season. During the first WNND outbreak, in 2008, the first reported case was in the 35th week; in 2016, the first horse that tested positive was reported in the 31st week; and in 2018, it was in the 26th week. The mean value of the seasonal period in these three years was at the 35th (2018) and 38th (2008, 2016) week. In the whole data set, the mean of the seasonal peak was at the 35th week (day 245). Table 2 contains the collective results of seasonality.

**Figure 2:**
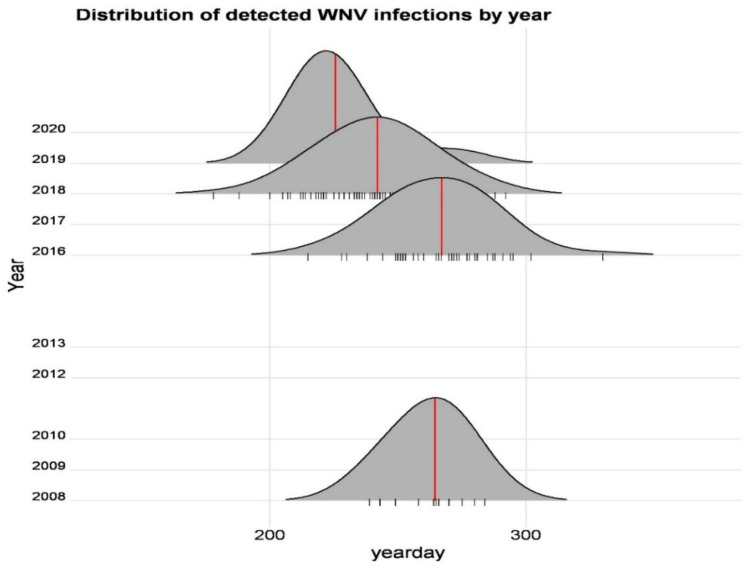
Distribution of detected WNV infections by year. Vertical lines along the x axis depict individual observations, the curve is a result of loess smoothing, to aid the visualization of the sample distributions, while annual median yeardays are marked with red lines on the diagram.

In Figure 2, the curves of each season can be seen. According to the sensitivity, the scale is divided by days. Displayed are only those examined years where information on more than five cases was available. 

### 3.3. Clinical Data

The age of the horses with acute confirmed WNND ranged from 3 months to 22 years of age, with a mean age of 8 years. The age of the horses was sorted into three age categories: 32.3% were under 5 years of age, 55.6% were between 6–15 years, and 11.3% were above 15 years old. Sixty-seven (54%) were male, while 49 (39.5%) were female. Apparent breed predisposition was not seen, seven different breeds were represented in the database. Most horses were Hungarian warmbloods (*n* = 84; 67.7%) and ponies (*n* = 17; 13.7%), but many other breeds such as Frisians (*n* = 8; 6.4%), Arabians (*n* = 6;4.8%), thoroughbreds (*n* = 4; 3.2%), draft horses (*n* = 4; 3.2%), and Przewalski horses (*n* = 1; 0.8%) were also affected. 

The most common clinical sign was ataxia, which appeared in a total of 81.5% (*n* = 101) of the cases. Lameness of any leg or hindquarter paresis (12.1%) often preceded generalized ataxia. Both fore and hind limb paresis and ataxia were recorded, but in many cases veterinarians could not distinguish between different types of ataxia. In the overall database, hind limb ataxia was most frequent (*n* = 40; 39.6%). General weakness was seen in many cases (62.1%; *n* = 77), which manifested in some of the cases as horses shifting their weight continuously from one leg to the other. Hyperaesthesia appeared in 35.5% (*n* = 44) of patients, and this was one of the most common and striking signs at the onset of the disease, and its severity ranged from very mild abnormality to sudden collapse on touching. Muscle fasciculation appeared to be a frequent clinical sign, in 37.9% (*n* = 47) of the examined patients. The triceps muscle was the most affected region, but muscle fasciculation could affect the face, hind limb, and thoracic muscles, or became generalized. Different clinical signs of cranial nerve deficits were observed in our study. Twenty-five horses (20.2%) were affected by temporary facial nerve paresis/paralysis. Nystagmus and dysphagia were only observed in a minor proportion; in 3.2% and 7.3% of horses studied. In 43.5% (*n* = 54) of the cases, behavioral changes were observed, including confused behavior, teeth grinding, and aggressive or self-harming behavior.

The most common initial clinical signs were lethargy and weakness, which appeared in about 62.1% (*n* = 77) of cases. Hyperthermia (over 38.3 °C up to 40.0 °C) was also a common sign at the beginning of the disease. Horses, mostly alongside fever, transiently showed loss of appetite, but generally, horses did not have anorexia after the initial phase, during their clinical progression. In 16.9% (*n* = 21) of the cases, the first abnormality was colic-like behavior, which appeared shortly before the onset of obvious neurologic signs, such as generalized weakness and ataxia. A total of 35.5% (*n* = 44) of horses became recumbent at some point of the disease progression. Clinicians also reported hindquarter paresis, with patients sitting in a dog position and unable to stand up without specific aids. Some horses could survive with prolonged recumbence if they were able to maintain a sternal position and kept on eating during the critical phase of the disease. Thirty-one (70.5%) recumbent horses did not survive, of which comatose conditions appeared in 16.1% (*n* = 5) of the cases. Horses with prolonged recumbency, collapse, and a comatose condition were euthanized on humane grounds in 61.9% of cases.

Table 3 contains the overall results and the annual distribution of each clinical sign. The signs are listed in descending order, according to their total frequency of occurrence. 

**Figure 3:**
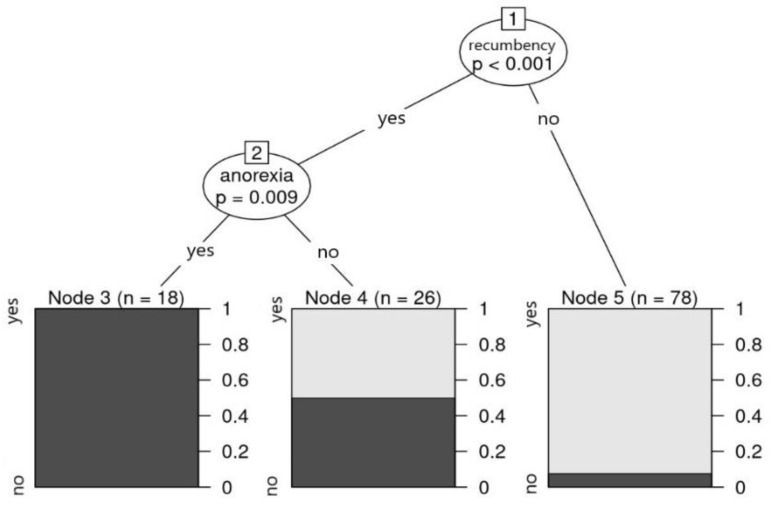
Decision tree for survival status according to all clinical signs. Recumbency and anorexia are two clinical signs that were able to categorize survival status. If both were present, the survival probability was 0 in our sample.

Information on outcomes was available for 122 of the 124 horses included in the clinical study. Overall, 68.5% (*n* = 85) of horses recovered and 29.8% (*n* = 37) died during the acute phase of the disease. During the biggest outbreaks, 68.6% (11/16) in 2008, 68.4% (13/19) in 2016, and 69.4% (50/72) in 2018 survived. The difference of survival probability among age categories was not significant (X2 = 1.77; df = 2; *p*-value = 0.4121). Moreover, 62.5% of the youngest 0–5 year old horses (25/40), 74.6% (50/67) 5–15 years old, and 71.4% (10/14) of the horses >15 years old survived. Foals (i.e., animals ≤ 1 year old) were more likely to die (66.7%; 6/9) as a consequence of WNV infection than older animals. The CART fitted on survival probability by symptoms showed that if recumbency and anorexia were both present, all individuals died. In the case of recumbency without anorexia, approximately 50% of the individuals survived, while the lack of recumbency resulted in an overall survival probability of 90%. In Figure 3, the CART results are displayed.

Follow up of recovery was only available in the case of 39 horses, due to the long data collection period. In the examination period between 2007 and 2013, 26.7% (4/15) of the affected horses still had residual signs at least 2 years after the recovery of the acute phase of WNND. After the 2018 outbreak, 25% (6/24) of the surviving equines had some residual sign related to the previous WNND. These signs were mild hind quarter ataxia (3/6), weakness (1/6), and behavior changes (2/6).

### 3.4. Treatment

**Figure 4:**
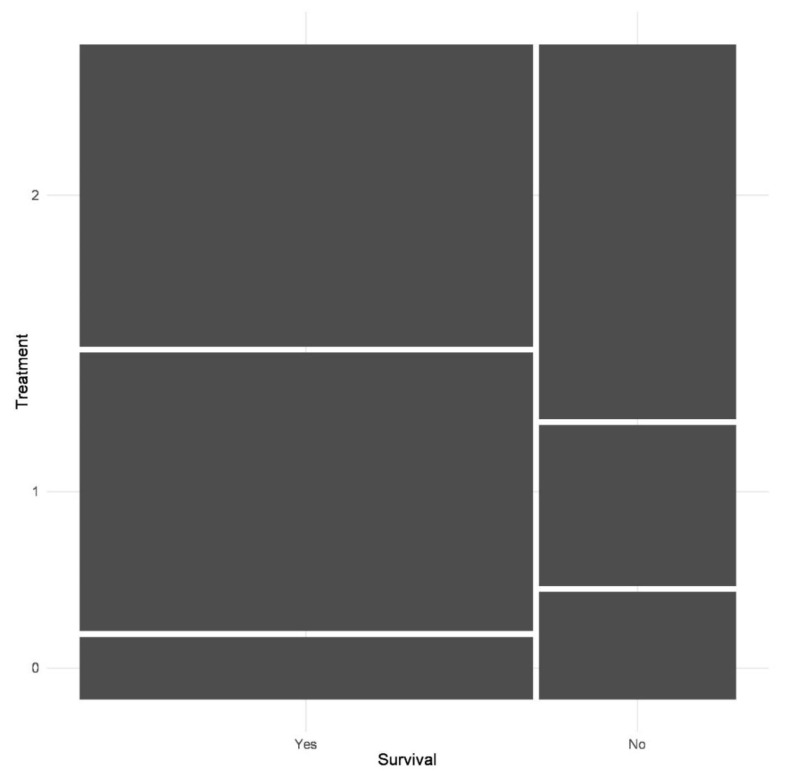
Mosaic plot of treatment protocols and the outcome of the disease (0 = no treatment; 1 = NSAID treatment, 2 = glucocorticoid treatment). The areas of the rectangles are proportional to the relative frequency of the given variable combinations. Mosaic plot for the correlation of treatment protocols and the outcome of the disease (0 = no treatment; 1 = NSAID treatment, 2 = glucocorticoid treatment).

Treatment protocols were divided into categories according to the administration of non-steroid anti-inflammatory drugs (NSAID) or glucocorticoids. In Group 0, (12.5%) the animals did not receive any medication during the disease. Group 1, including 46 horses (37.1%), were treated with NSAID drugs; while in the Group 2, with 48.4% of all patients, horses received glucocorticoid drugs at least once during the acute phase of the disease. We analyzed the outcome of the disease in connection with treatment protocols. There was no significant correlation shown by the Pearson’s Chi-squared test (X2 = 3.93; df = 2; *p*-value = 0.1397) between the treatment protocols and the survival rate of the WNND in horses. The results are displayed in Figure 4. 

## 4. Discussion

We provide a comprehensive report on the Hungarian West Nile virus infections of equids from the first detection, through the massive transmission seasons in 2008, 2016, and 2018, until 2020. Our study includes the temporal and geographical distribution of 198 horses tested positive for WNV IgM, and the clinical description of 124 WNND cases, with information on the general data and clinical signs of the affected equines. 

The risk of a WNV outbreak is multifactorial; the viral, host, and environmental circumstances all need to be taken into account. In our study, covering 13 transmission seasons, all the CNS samples tested for PCR proved to be lineage 2 strains; during multi-approach research, trapped mosquitos adjacent to equine WNV cases proved to be infected with the same lineage 2 strains [43,44]. These lineage 2 strains served as the origin of several epidemics across Europe (Italy, Austria, France, Germany, and the Netherlands) [14,15,16,45,46]. The epidemiology of these European lineage 2 strains is similar to the lineage 1 strains, which were detected in North America and spread all over the American continent. However, lineage 1 strains also circulate in Europe, but they cause geographically and temporally localized outbreaks in Spain, Italy, and France, and do not spread to more distant regions [26,45]. Hungary reports human and equine WNND cases every year, and the whole country became endemic during recent years. The same pattern in incidence and geographic distribution can be seen in both humans and horses [44,47]. Significant outbreaks during the 2016 and 2018 transmission seasons drew attention to this pathogen and to the fact that the occurrence of new outbreaks was largely unpredictable. On the other hand, we might need to take population seropositivity into account. After the 2018 outbreak in Hungary, we detected 66.5–72.3% seropositivity in two different studies, on 236 and 112 horses, respectively (unpublished data), which might partly explain why case numbers decreased in the subsequent years, to 11, 1, and 3, in 2019, 2020, and 2021, respectively (ECDC). This suggests the hypothesis that, after a massive outbreak, individuals of different species might become asymptomatically seropositive and, as such, limit the spread of the virus. Considering the geographical spread, newly infected areas may also occur in relation to the vector and host density, and their interactions. The Hungarian capital and the surrounding areas were heavily affected during all outbreaks. Population density can explain the this observation, but it could be also due to economic reasons. Horse owners around the capital have better access to veterinary care, information, and diagnostic techniques. Hajdú-Bihar County and the area of Hortobágy are well known for their migratory avian population, which carries a continuous risk of new virus introductions from Africa and where the bird population density also favors virus transmission [48,49]. Overwintering of mosquito species and temperature changes strongly correlate with virus enrichment in optimal areas [48]. Changes in diversity play an important role, not only in terms of territory, but also in terms of seasonality. If many migratory birds leave in the autumn, the viremia may increase in the remaining birds, and thus the density of the affected and susceptible species and the infestation of these secondary species (birds, humans, horses alike) may increase [50]. The seasonal appearance of WNV infection has a strong parallel relationship with the vector activity and climate circumstances in the observed area [48]. Although the seasonal onset of WNV infections has appeared earlier than previously, the highest incidence rate is still between August and October. It is important to consider the current seasonal variations when developing vaccination strategies. Since the virus spreads from year to year to new European areas, mainly in the northwest, it is recommended to include WNV vaccines in vaccination protocols, even in non-endemic areas of Europe; especially in the case of horses traveling to known endemic areas. 

A publication on human WNND stated that the risk of developing a severe neurological form of the disease is higher in the elderly [25]. A similar pattern of relative risk according to age group could not be demonstrated in our study. The mean age of horses was 8 years old, and other studies describing equine outbreaks published similar findings, with mean ages of 6.9 [51], 8 [31], and 9.5 years [28]. The reason why middle-aged horses are overrepresented in most of these studies is multifactorial. On one hand, this might reflect the age distribution of the horse population; on the other hand, foals, yearlings, and retired older horses are usually kept on pastures with less surveillance, so cases might be undetected. We found nine affected foals under 12 months of age, with a fatality rate of 66.7%, and these results suggest that younger animals are less likely to survive. In some other studies, a higher mortality rate was observed in the older population, with mean age of 10.8 ± 7.8 years [28]. 

Hungarian warmblood horses appear to be somewhat overrepresented in the present study, and other breeds appeared to be underrepresented. Breed predisposition reflected the composition of the Hungarian horse population rather than a predisposition to infection, and due to small numbers of individual breeds, statistical associations could not be made. The same observation regarding local breed predisposition was also mentioned in other studies [27,28].

Previous studies in humans and horses have shown that males are more likely to develop neurologic forms than females. In the present study, male equids were also overrepresented, but we could not demonstrate a significant association [14,25,26,27,28,52]. 

In human medicine, several manifestation categories have been described for WNV infections, such as WN Fever, WN Meningitis, WN Encephalitis, and WN Poliomyelitis [25]. The same classification cannot be used in horses, as the signs are more complex and the diagnostic possibilities are less sensitive for these kinds of differentiations. Fever, depression, and anorexia are considered general immune system activities and occur during, or immediately after, the viremia phase [25,27]. Horses may only be febrile for a short period, likely early in the disease, and fever may be associated with viremia. These cases of West Nile fever clinical presentation possibly go unnoticed, where short-term hyperthermia or depression is not detected and only horses with more severe signs receive veterinary care. West Nile fever without any other neurologic sign can occur in equines, based on the observations of a previous study, where WNV IgM-positive tested horses had only fever [32]. In our study, we only used the clinical data of horses with acute neurologic signs and might have missed cases of West Nile fever.

The clinical manifestation of WNND in horses reported in the present study was similar to those observed in previous publications, and ataxia was the most common clinical sign [10,11,14,26,27,28,29,31,34,51,52]. Although both lineages cause gait incoordination, our study and other publications on lineage 2 [11,14,30] observed a higher, 100% and 82%, presence of ataxia compared to studies describing lineage 1 outbreaks, with an occurrence rate of 57% and 69% [28,31]. Both fore- and hindlimb ataxia can be observed during outbreaks, and the ataxia can be pronouncedly asymmetrical and can also affect all four limbs [10]. Primary forelimb ataxia is repeatedly described in lineage 2 outbreaks [10,14], but may also occurs in lineage 1 cases [28]. Other movement disorders may appear in various forms, from mild lameness to severe paresis. Both weakness and paresis are characteristic neurological signs of the disease, affecting 62% of our patients; this observation is consistent with the findings of other publications [28,30]. Ataxia and weakness are signs of brain and spinal cord disease. They may appear as part of a direct infection of the spinal cord, interruption of motor tracts in the hindbrain, or loss of fine motor control, with the infection of the large nuclei of the thalamus and the basal ganglia [27]. Weakness has been found to progress to recumbency in approximately one-third of cases. When no loss of consciousness occurred and the recumbent horses were forced to stand up with the help of slings or at least kept in sternal recumbency, the survival rate increased.

In one equine patient, a progressively worsening hindquarter paresis was observed 2 months after recovery from mild neurological WNV disease during the 2018 epidemic season. In humans, it has been described that a worsening of nervous system signs may appear in the long term, which are presumably not caused by the presence of the primary virus, but rather have an immune-mediated origin. Paresis or paralysis may be part of the acute disease, but we hypothesize that these later signs are part of an immune-mediated reaction. The affected horse survived the disease and without histopathological examination, this hypothesis cannot be confirmed.

Hyperesthesia and muscle fasciculation were frequently detected signs, and their incidence rate was above one-third of the cases. The localization of these severe signs in our study was similar to that described by others [14,28,29,31]. In human cases, muscle fasciculation has also been mentioned as part of West Nile encephalitis [25], and it can form a diagnostic criterion in horses with WNV encephalomyelitis. The pathogenesis of this abnormality likely includes loss of fine motor control [27].

Equine Herpes Myeloencephalitis (EHM), which is also a relatively common cause of neurologic signs in horses in this geographic region, should be considered as a differential diagnosis. The clinical signs are well-differentiated in some cases, such as the lumbosacral signs of EHM (hind limb ataxia, weakness, bladder paralysis) being very different from those induced by the brainstem damage of horses with WNV infection (four-limb ataxia, muscle fasciculation, hyperaesthesia), but there may be overlaps in the clinical picture in some cases, which is why background laboratory tests are essential for differentiation. Epidemiological features, such as seasonality or the co-morbidity of several horses in the same place, may also be helpful in distinguishing EHM from WNND.

Even though facial nerve paralysis, dysphagia, and nystagmus are considered common signs of WNND, they appeared to be scattered during our study. In the absence of histopathologic examinations, we cannot declare for sure the exact location of brain injuries, but according to previous publications, histologic lesions within the pons and medulla oblongata can explain the clinical deficits of cranial nerves. Other publications on lineage 2 WNND mentioned facial nerve paralysis occurred twice as often, as we experienced [14], although the appearance of this sign differs in a wide range in other publications [27,28,52]. Dysphagia and nystagmus were uncommon and were reported with the same incidence as in other countries [27,28].

Colic-like behavior was a common initial and conspicuous sign of the disease. This sign often appeared first and persisted for a short time, before the severe neurological disorders occurred, and sometimes had a misleading effect on the veterinary examination in field circumstances. Our reported gastrointestinal signs are consistent with previous observations of equine epidemics [14,27]. Colic-like behavior can be a behavioral disorder that appears as a central nervous system sign, but also can appear as real colic with abdominal pain, which is caused by an injury to the autonomic nervous system. In hamster models, WNV was successfully isolated from the myenteric neurons. Increased contrast retention in the stomach compared to control hamsters supported the observation that gastrointestinal muscles may receive less nerve stimulation from the myenteric plexus [53]. In addition to gastrointestinal dysfunction, damage to the autonomic nervous system is also supported by the observation that heart rate variability (HRV) diminished and cardiac arrhythmias occurred in WNV-infected hamsters and humans [25,53]. HRV is an appropriate clinical marker of autonomic dysfunction. Different types of behavior changes frequently illustrate the involvement of different parts of the brainstem [25,27]. In addition to colic-like behavior, self-harming, aggressive behavior, narcolepsy, and deep depression were noticeable signs in our study. Others also mentioned these disorders during the acute phase of WNND or as residual signs [14,27,28].

Although we could not significantly detect it in the results, Figure 3 shows that glucocorticoid-treated animals were less likely to survive the disease. This observation is not consistent with the previous description of lineage 1 epizootic, where Porter et al. published opposite results [24]. There may be several reasons for this difference, but it is likely that the relatively low number of cases in both studies impairs successfully making an accurate comparison. The timing of glucocorticoid administration may also have affected the results. In our study, horses were mostly given this type of medication when CNS signs were rapidly progressing and leading to severe weakness or recumbency, and it can be assumed that administration of glucocorticoids earlier during the disease progression might have increased its efficacy. On the other hand, our results might reflect a real negative effect on the survival, and this may even have worsened the condition of patients by increasing viremia, as was shown in dogs [54]. We cannot claim that WNND has a specific treatment, but we observed that an infection detected at an earlier stage and a well-organized supported therapy may have a beneficial effect on the outcome.

Collective survival rates differed in a wide range, mostly depending on the number of examined equines, rather than on the lineage. Comparing our findings to studies with a large number of cases, the fatality rate remained relatively at the same level, around 25–30% [27,28]. We cab hypothesize that descriptive publications on local outbreaks with a small number of cases, instead, show the most severe cases, with a higher rate of poor prognosis [14,30]. On the other hand, almost all studies stated that recumbence seems to be the best marker of fatal outcome, an observation that concurs with our findings. Furthermore, we found that horses without a loss of appetite and with adequate support could be fed in a recumbent position and had a higher chance of survival. In the context of this observation, we assume the more severe brainstem lesions lead to a fatal outcome. Another remarkable observation is that approx. 70% of the deaths were euthanized for humane reasons, which is also mentioned in most other reports. The background of this high amount of euthanasia should be taken into account on a case-by-case basis, and usually it is a consequence of a poor prognosis with a long, costly treatment.

## 5. Conclusions

West Nile virus has been an endemic pathogen in Hungary for the last 14 years, causing severe outbreaks among the equine and human population all over the country. Owing to their higher susceptibility, the examination of equines may play an important role in predicting outbreaks. An overall surveillance system in Hungary that includes both disease and PCR screening results would provide important information for both veterinary and human medicine. WNV infection is recognized in time, and a well-organized supported therapy is an essential factor for the successful outcome of the disease. Future studies may use equine cases as an indicator of WNV-intensive transmission activity and epidemiological and entomological studies to further understand the risk factors of WNV epidemic transmission. The collective results of this article can allow a comprehensive overview of the Hungarian Equine WNND cases and serve as the base point for collaborative inter-discipline research and programs on West Nile virus. Our work also reflects the urgent need for a national, organized surveillance system related to West Nile virus within the One Health approach.

## 6. Limitation

Our results were based on the passive surveillance system of the Hungarian National Food Chain Safety Office (NÉBIH) and examined a broad period. The exact onset of signs may differ from that reported, due to the reactions of horse owners. Many veterinarians carried out clinical examinations all over the country, and despite the same clinical examination protocol, differences may appear in the evaluation of clinical trials.

## Figures and Tables

**Table 1:** Number of clinically exanimated animals by year, and distribution in the whole dataset.

Year	2007	2008	2010	2012	2013	2016	2017	2018	2019	Total
Examined number of horses (*n*)	2	16	2	1	4	19	2	72	6	124
Percentage of cases according to year (%)	1.6	12.9	1.6	0.8	3.2	15.3	1.6	58.1	4.8	100

**Table 2:** Collective seasonal data of the whole dataset.

Year	2007	2008	2009	2010	2012	2013	2016	2017	2018	2019	2020
*n*	2	18	1	2	1	4	47	3	109	10	1
mean (week)	44	38	33	30	40	36	38	35	35	32	38
median (day)	306.5	264.5	229	213	275	244	267	253	242	225	261
first case (day)	306	239	229	213	275	213	215	219	178	214	261
last case (day)	307	284	229	213	275	289	330	253	292	271	261

**Table 3:** Results of clinical signs.

Clinical Sign	Total Number (n)	Total Percentage (%)	2008 (n; %)		2016 (n; %)		2018 (n; %)	
ataxia	101	81.5%	11/15	73.3%	15/19	78.9%	60/70	83.3%
lethargy/depression	77	62.1%	11/14	78.6%	10/19	52.6%	45/72	62.5%
paresis/weakness	77	62.1%	10/11	90.9%	8/19	42.1%	47/72	65.3%
behavior change	54	43.5%	9/12	75.0%	11/19	57.8%	20/72	27.8%
anorexia	51	41.1%	6/14	42.9%	7/19	36.8%	29/72	40.3%
hyperthermia	50	40.3%	3/15	20.0%	8/19	42.1%	31/72	43.1%
muscle fasciculation	47	37.9%	8/12	66.7%	5/19	26.3%	30/72	41.7%
recumbence	44	35.5%	7/12	58.3%	7/19	36.8%	27/72	37.5%
hyperesthesia	44	35.5%	5/11	45.5%	6/19	31.6%	27/72	37.5%
n. facialis paralysis	25	20.2%	8/13	61.5%	3/19	15.7%	8/71	11.3%
colic	21	16.9%	2/15	13.3%	6/19	31.6%	10/72	13.9%
limb paralysis	16	12.9%	3/11	27.3%	2/19	10.5%	10/72	13.9%
lameness	15	12.1%	0/16	0.0%	8/19	42.1%	4/72	5.6%
dysphagia	9	7.3%	1/15	6.7%	1/19	5.3%	4/72	5.6%
nystagmus	4	3.2%	0/14	0.0%	0/19	0.0%	2/72	2.8%

## Data Availability

Restrictions apply to the availability of these data. Data was obtained from Veterinary Diagnostics Directorate of Hungarian National Food Chain Safety Office (NÉBIH) and are available with the permission of the Veterinary Diagnostics Directorate of Hungarian National Food Chain Safety Office (NÉBIH).

## Supplementary Materials

The following supporting information can be downloaded at: https://www.mdpi.com/article/10.3390/v14112551/s1, Figure S1: Standard examination questionnaire.
