# Supplementary material for: Epidemiology and Clinical Manifestation of West Nile Virus Infections of Equines in Hungary, 2007–2020
Source: Viruses. 2022 Nov 18;14(11):2551. doi: 10.3390/v14112551 (PMC9694158; doi:10.3390/v14112551)
Supplement: Supplementary file 1 [file viruses-14-02551-s001.zip › viruses-1975301-supplementary.pdf]

Clinical examination questionnaire

Official registered number at NÉBIH:
Veterinarian:
Onset of disease:
Sampling date:
Place, county:

General data of equine:

- Age:
- Gender: Female /Male
- Usage: Sport / Hobby / Breeding / No usage
- Stable circumstances: closed stable / field / both
- Vaccination history:
- Previous illnesses:

Clinical signs:

- Hyperthermia
- Anorexia
- Depression
- Behavior change
- Hyperesthesia
- Muscle tremors
- Muscle fasciculation
- Ataxia
- Weakness / Paresis
- Paralysis
- Lamness
- Recumbence
- Colic
- Dysphagia
- Nystagmus
- Cranial nerve paralysis

Treatment:

- NSAID
- Dimethyl- Sulfoxide
- Glucocorticoids
- IV fluid
- Others:

Outcome: survived / died – euthanized

Figure S1. Standard examination questionnaire.
